# Entomopathogenic fungus *Akanthomyces muscarius* (Hypocreales: Cordycipitaceae) strain IMI 268317 colonises on tomato leaf surface through conidial adhesion and general and microcycle conidiation

**DOI:** 10.1080/21501203.2021.1944929

**Published:** 2021-07-05

**Authors:** Oumi Nishi, Hirotoshi Sushida, Yumiko Higashi, Yuichiro Iida

**Affiliations:** National Agriculture and Food Research Organization (NARO), Mie, Japan

**Keywords:** *Lecanicilliummuscarium*, *Verticillium lecanii*, epiphyte, mucilage, integrated pest management, trichome

## Abstract

The entomopathogenic fungus *Akanthomyces muscarius* strain IMI 268317, previously known as *Lecanicilliummuscarium*and *Verticillium lecanii*, is currently used as a microbial insecticide to protect tomatoes from serious leaf-inhabiting pests in greenhouses. However, its persistence on tomato leaves has been unidentified. Understanding the events and processes of phyllosphere colonisation by this strain should help in developing its practical applications. This study assessed the epiphytic abilities of this strain on tomato leaves in humid conditions, simulating closed greenhouse environments. Conidia applied on tomato leaflets strongly adhered 12 h after inoculation. The mucilage-like materials were found around the germinated conidia after 3 days after inoculation (dpi), which possibly strengthened the adhesion. A total of 15% of conidia germinated at 3 dpi, of which 2% formed typical conidium or an enlarged structure on germ-tube tips. Many conidia were produced on phialide tips that branched from elongated hyphae at 7 dpi; however, invasion into leaf tissue was not observed. On the leaflets, inoculated conidia suspensions of 1 × 10^5^ and 1 × 10^6^ conidia/mL, colony forming units increased 52.6 and 8.8 folds from 0 to 14 dpi, respectively. These results suggested that *A. muscarius* strain IMI 268317 has high epiphytic abilities on tomato leaflets in a humid condition.

## 1. Introduction

Mitosporic hypocrealean fungi such as *Akanthomyces* spp. Lebert, previously known as *Lecanicillium* spp. (W. Gams and Zare) (Hypocreales: Cordycipitaceae), *Beauveria bassiana* (Bals.-Criv.) Vuill. (Hypocreales: Cordycipitaceae), and *Metarhiziumanisopliae* (Metsch.) Sorok. (Hypocreales: Clavicipitaceae) have been developed as biocontrol agents against a wide range of insect pests (Faria and Wraight 2007). These fungi have been frequently found as natural epiphytes, endophytes, and rhizosphere colonisers as well as insect parasites (Meyling and Eilenberg 2006; Behie et al. 2015; Garrido-Jurado et al. 2015). As expected from this nature, some isolates of these species were established as plant associates; However, in some case, the establishments were transient (Posada and Vega 2005; Quesada-Moraga et al. 2014; Klieber and Reineke 2016; Resquín-Romero et al. 2016; Garrido-Jurado et al. 2017; Jaber and Ownley 2018). Because some of these fungi can also protect plants from infection of nematodes and pathogens (Shinya et al. 2008; Vega et al. 2008, 2009; Bamisile et al. 2018), they play multiple roles in integrated pest management strategies for sustainable crop production.

Several pests were known to inhabit tomatoes, of which spider mites, thrips, whitefly, and leaf miner fly develop resistance against many chemical pesticides (e.g. Kliot et al. 2016). Therefore, new management strategies are needed for these pests. Entomopathogenic fungi and predatory insects have been alternatively used to control these pests. Secretions from tomato plant surface restrict some important predatory mite activities (Sakamoto et al. 2012); thus, high expectations are specifically placed on entomopathogenic fungi for biological control tomato pests. Additionally, epiphytic or endophytic entomopathogenic fungi may efficiently control these leaf-inhabiting pests (Klieber and Reineke 2016; Barta 2018).

The common approach, the use of the biocontrol agents, was used wherein spore suspensions are directly sprayed onto plant leaves or stems, resulting in a temporarily high concentration of fungal spores on plant surfaces. *Akanthomycesmuscarius* IMI 268317 (also known as Ve6, CBS 102.071, and ARSEF 5128)is used as an active ingredient of the commercial microbial insecticide that controls tomato pests in greenhouses (cf. Product information of Mycotal® fromKoppert Biological Systems Ltd. or ArystaLifeScience Co., European Food Safety Authority 2010); however, its persistence on tomato plants has been unidentified. Understanding the events and processes of the phyllosphere colonisation of this strain should help in developing practical applications. In this study, we assessed the epiphytic abilities of the strain IMI 268317 on tomato leaves in humid conditions, simulating closed greenhouse environments.

## 2. Materials and methods

### 2.1. Fungal isolates

*Akanthomycesmuscarius* strain IMI 268317, a single-spored isolate from the commercially available biocontrol product of *A. muscarius* (Mycotal®, ArystaLifeScience Co., Tokyo, Japan), was used throughout the study. For validity of species identification of this isolate, DNA sequence of the ITS1-5.8S rDNA-ITS2 region of this isolate was confirmed to be close (identity = 498/500, gap = 2/500) to that of *A.muscarius*IMI 068689 (ex-epitype strain).For the confocal laser scanning microscopy (CLSM) study, the strain was transformed with pFT1 harbouring green fluorescent protein (GFP) gene placed downstream of a ToxA promoter and Hygromycin B resistance gene by an *Agrobacterium*-mediated transformation. The pFT1 vector was provided by Gert Kema (Plant Research International, Wageningen, the Netherlands). The transformants were examined using a confocal laser microscope (LSM 700, Carl Zeiss, Oberkochen, Germany). Images were acquired by excitation with 488 nm argon laser and using a filter (495–515 nm) to detect fluorescence emitted by the transformant using the LSM software ZEN 2010 (Carl Zeiss, Oberkochen, Germany). One of the transformants showing appropriate GFP fluorescence emission and stability was selected to carry out bioassays. Monosporic cultures of the wild and GFP-transformed strain, known as Amgfp, were grown on modified Sabouraud dextrose yeast extract agar (SDYA; Glucose, 20 g/L; Peptone, 2 g/L; Yeast Extract, 2 g/L; Agar, 15 g/L) at 25°C in the dark, stored at 4°C.

### 2.2. Plant material

Seeds of the commercial tomato *Solanum lycopersicum* cv. Regina provided by the Sakata Seed Co. (Kanagawa, Japan), were used throughout the study. Seedlings were grown from seeds into a soil mixture (NippiEngeiBaido 1, Nihon Hiryo Co., Tokyo, Japan) in soft polyethylene pots (ø75 × 68 mm) and incubated in a phytotron at 25°C, and 16/8 h light/dark photoperiod. Fertiliser was originally mixed in the soil mixture and not added after plantation. The seedlings were not washed before bioassays because no visible soil particles were observed on plant surface.

### 2.3. Preparation of conidial suspension

To obtain a conidial suspension, the *A. muscarius* strain IMI 268317 or the transformant Amgfp were grown on SDYA plates for 7–14 days at 25°C–27°C in the dark. Conidial suspensions were prepared by scraping them from the plates into a 40-mL aqueous sterile solution of 0.05% Tween 20, filtered through a sterile plugged funnel with gauzes to remove hyphae. The suspensions placed in 50-mL centrifuge tubes were washed twice with the same solutions after centrifugation for 5 min at 1000 g at 20°C. Conidia concentrations were determined using a haemocytometer, and appropriate dilutions were made in 0.05% Tween 20 to obtain an adjusted suspension.

### 2.4. Adherence assay of conidia on tomato leaflets

A tomato leaf containing five leaflets was cut off from 6–8 week-old tomato plants and placed in a square dish (140 × 100 × 14.5 mm) lined with a wet cotton sheet. A drop (2 µL) of conidial suspension adjusted to 5 × 10^5^ conidia/mL (1000 conidia/drop) was placed on the adaxial surface of the leaflets. The square dish was then placed in a plastic box (241 × 331 × 190 mm). The inner surface of the box was moistened by spraying distilled water to maintain near-saturated to saturated humidity (95%–100% RH). Leaflets inoculated with a conidial suspension were punched at 0- and 12-h post-inoculation (hpi), and leaf disks (8 mm diameter) were carefully placed in a well of a 48-well plate, not to disturb the drop.

A hollow cylinder cut off from a micropipette tip was placed on the leaf disk as a weight to prevent the disk from floating during the washing procedure. The inoculation point on the leaf disk was washed by pouring a sterile aqueous solution of 0.05% Tween 20 (500 µL) onto the leaf disk and pipetting 10 times. The solution was transferred to a 1.5 mL tube, and the washed leaf disk was removed from the well. For collecting the remaining conidia in the well, a new 500 µL of a sterile aqueous solution of 0.05% Tween 20 was poured in the same well and successively transferred to the same 1.5 mL tube.

The washed leaf disk was transferred to a mortar and ground for 30 s after adding a 500-µL sterile aqueous solution of 0.05% Tween 20. The homogenised suspension was transferred to a new 1.5 mL microtube. To collect the remaining conidia in the mortar, a new 500-µL sterile aqueous solution of 0.05% Tween 20 was poured in the mortar and the suspension was successively transferred into the same 1.5 mL microtube.

The collected suspension was centrifuged (1000 × g, 5 min, 20°C). An aliquot of the supernatant was removed, and the remaining suspension (100 µL) was spread on a selective SDYA (modified SDYA, amended with hygromycin 0.1 g/L and chloramphenicol 0.1 g/L). Hygromycin was added to the medium to detect only the transformant harbouring hygromycin-resistant gene. CFUs were counted 4 days after plating.

### 2.5. Inoculation and quantification on tomato leaf

Tomato plants that are 6–8 weeks old and grown in a phytotron were used for the experiment. The 1 × 1-cm regions were marked on the leaflets using a permanent marker (four points on the vertexes) before inoculation. The plants were sprayed with conidial suspensions of the transformant (1 × 10^5^ or 1 × 10^6^ conidia/mL). Plants treated with an aqueous sterile solution of 0.05% Tween 20 served as the control treatment. The suspensions were inoculated on the whole above-ground plant tissues with a manual hand sprayer. Plants were placed in a plastic box with near-saturated to saturated humidity (95%–100% RH).

Four squares (inner regions of the four vertexes marked before inoculation, whose areas become larger than 1 cm^2^ at 7 and 14 days after inoculation (dpi) due to outgrowth during the period) were trimmed from the leaflets and immersed in a 1.5-mL volume microtube containing 1 mL of sterile distilled water containing 0.05% (v/v) Tween 20 at 0, 7, and 14 dpi. The tubes were vigorously vortexed for 5 min, and a dilution series was spread on selective SDYA and incubated for 2 weeks at 25°C. Population numbers (colony forming units: CFUs) of Amgfp on the leaf surfaces were estimated. The experiment was repeated thrice.

### 2.6. Observation by scanning electron microscopy (SEM) and CLSM

A tomato plant was inoculated with a conidial suspension of 1.0 × 10^7^ conidia/mL. Leaf disks trimmed from the inoculated plants at 1, 2, 3, and 7 dpi were fixed in a 2.5% (v/v) glutaraldehyde in a 0.1-M sodium cacodylate buffer (pH 7.2) for more than 24 h at 4°C. The samples were dehydrated using a graded series of ethanol concentrations (50%–100%), followed by immersion in a 100% tert-butanol. Specimens were coated with gold-palladium (20:80) in a Polaron E5100 sputter coating unit (Polaron Equipment, Hertfordshire, UK). Photographs were taken with a JEOL JSM-35 SEM (JEOL Co., Ltd., Tokyo, Japan) at 20 kV.

The proportion of the germinated conidia and conidia with a swollen germ-tube tip or microcycle conidia at 3 dpi was calculated. Nine fields of view containing 264–298 conidia were examined.As an inert surface control of leaf surface, conidial suspension inoculated on slide glass and incubated in a plastic box with near-saturated to saturated humidity (95%–100% RH) was observed at 3 dpi with a phase contrast microscope. One field of view containing 212–248 conidia was examined. The two experiment were conducted in triplicates.

The colonisation of Amgfp on tomato leaflets was also observed using the CLSM at 1, 3, 7, and 14 dpi to confirm the viabilities of identified fungal structures. Green fluorescence of GFP indicates the viability of fungal cells. To detect fungal propagules in leaf tissues, leaflets were excised from the inoculated plants at 3 dpi, hand-cut into thin slices with a razor blade, and their cross-sections were observed using CLSM. Images acquired were described in 2.1.

### 2.7. Statistical test

For the adherence assay, the total CFUs (remaining CFUs + CFUs washed away from leaflets) and adherence rates (remaining CFUs/total CFUs × 100) (%) were compared between 0 and 12 hpi. Independent triplicate experiments were conducted, each with three sampling replicates (i.e. three leaf disks from one plant). The pooled datasets of the triplicates (n = 9) were compared between 0 and 12 hpi by Wilcoxon’s rank-sum test using the R version 3.4.3 (www.r-project.org/).

For epiphytic colonisation assay, CFUs/cm^2^ were compared among 0, 7, and 14 dpi. Independent triplicate experiments were conducted, each with four sampling replicates (i.e. four 1 × 1 cm^2^ squares from different leaflets). The pooled datasets of the triplicates (n = 12) were compared by Wilcoxon’s rank-sum test. *p*-values were adjusted for the multiple comparisons in accordance with Holm’s method using the R version 3.4.3.

## 3. Results

### 3.1. Adherence of conidia on tomato leaf surface

**Figure 1.**
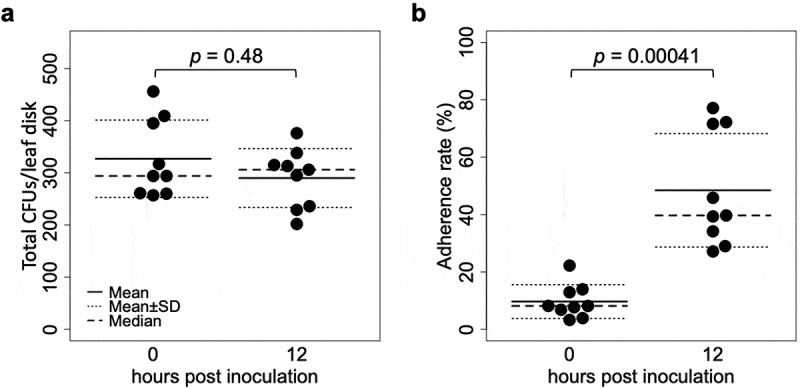
CFUs detected from tomato leaflets inoculated with a GFP-expressing strain Amgfp derived from *Akanthomyces muscarius* IMI 268317. (a) Total CFUs detected from leaf disk inoculated with a conidial suspension drop. (b) Proportion of CFUs detected from washed leaf disk.

The CFUs washed away from inoculated leaflets, and those that remained on leaflets after the wash (10 times pipetting) were quantified at 0 and 12 hpi. Although the total CFUs did not differ between 0 and 12 hpi (n = 9, *p* = 0.48, Figure 1(a)), the washed CFUs decreased from 297.8 ± 82.1 to 151.8 ± 73.5 (mean ± S.D., n = 9, *p* = 0.0027) and the remaining CFUs increased from 29.2 ± 14.4 to 138.2 ± 56.9 (mean ± S.D., n = 9, *p* = 0.00041). Therefore, the proportion of the remaining CFUs (i.e. adherence rate) also increased from 9.7% ± 8.1% to 48.5% ± 39.7% (mean ± S.D., n = 9, *p* = 0.00041) (Figure 1(b)).

### 3.2. Epiphytic growth on tomato leaf surface

**Figure 2.**
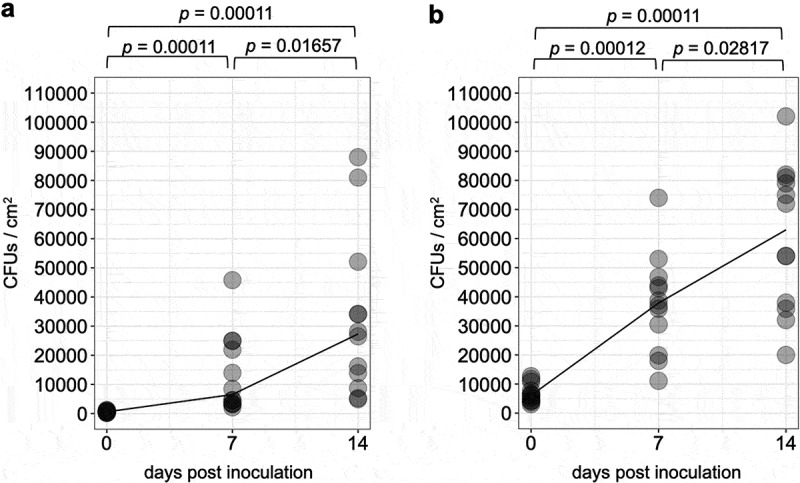
The number of colonies forming units over time detected on tomato leaflets treated with conidial suspension of 1 × 10^5^ (a) or 1 × 10^6^ (b) conidia/ml of a GFP-expressing strain Amgfp derived from *Akanthomyces muscarius* IMI 268317. Lines indicate medians.

CFUs on inoculated leaflets continuously increased over time for both the two doses of inoculation (Figure 2(a), b, n = 12, adjusted *p* < 0.05). On leaflets inoculated with 1 × 10^5^ and 1 × 10^6^ conidia/mL of conidia suspensions, CFUs increased 52.6 and 8.8 folds from 0 to 14 dpi, respectively, when their average values were compared.

**Figure 3.**
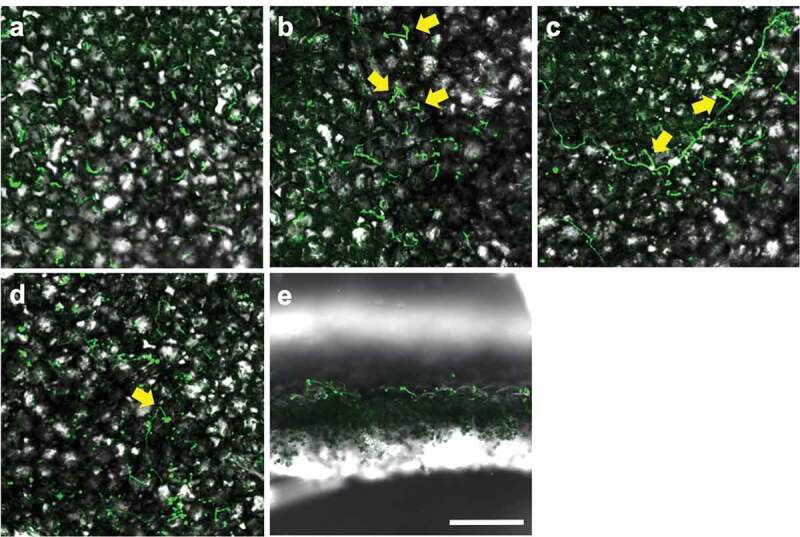
Laser scanning confocal microscopy images of epiphytic colonisation of a GFP-expressing strain Amgfp derived from *Akanthomyces muscarius* IMI 268317 on tomato leaflets. Viable conidia and hyphae were detected on tomato leaflets surfaces (a–d) and cross section (e) at 1 dpi (a), 3 dpi (b and e), 7 dpi (c), and 14 dpi (d). Microcycle conidiation like structures in (b) and phialides with conidia (c and d) were arrowed. Scale bar = 100 µm.

**Figure 4.**
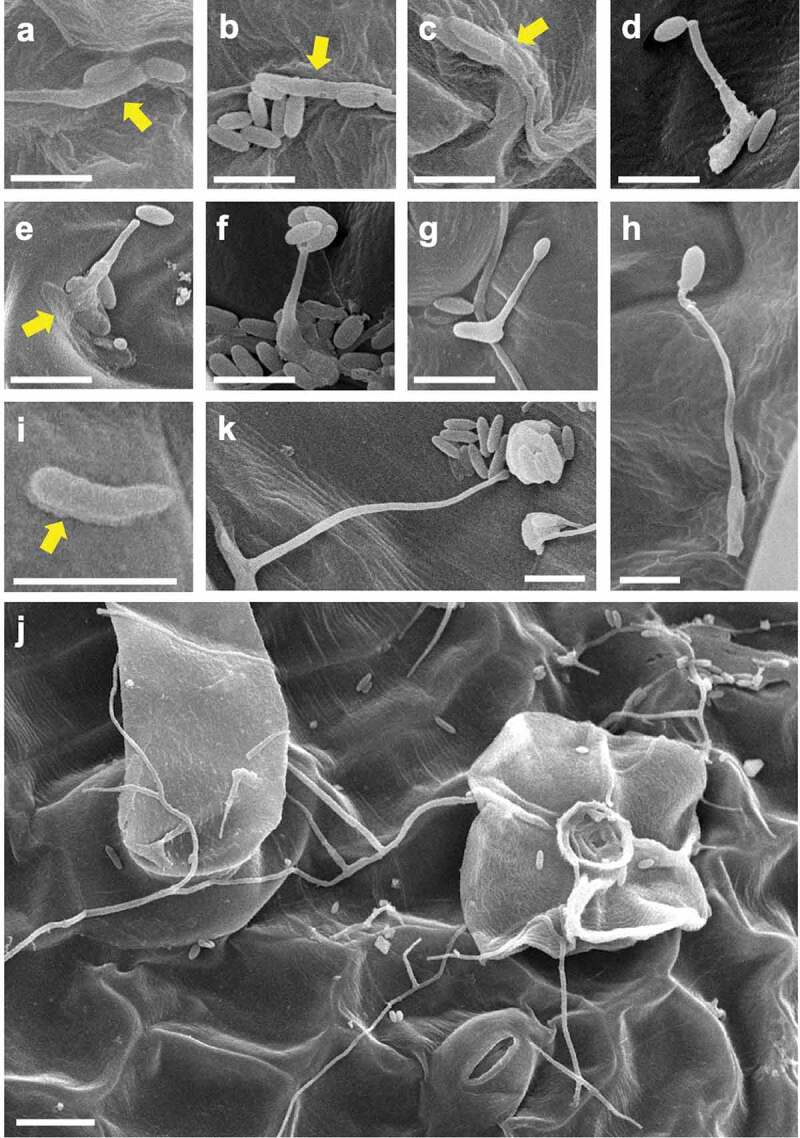
Scanned electron microscopy images of epiphytic colonisation of a GFP-expressing strain Amgfp derived from *Akanthomyces muscarius* IMI 268317 on tomato leaflets at 1 dpi (a–c), 3 dpi (d, f–i), and 7 dpi (e, j, k). (a–c) ambiguated boundaries between germinated conidia and adjacent conidia or leaf surface, (d) a germ-tube with a conidium on tip, (e) a germ-tube with a conidium adjacent to a tip, and mucilage-like materials around a conidium (arrow), (f) multiple conidia on germ-tube tip, (g) a germ-tube with a swollen tip, (h) a germ-tube with an appressorium-like swollen tip, (i) a germinated conidia with mucilage-like materials (arrow), (j) conidia and elongated hyphae around a trichome, (k) a mass of conidia produced on a tip of a solitary phialide. Scale bar, 5 µm (a–i, k), 10 µm (j).

CLSM and SEM observation of Amgfp on tomato leaves revealed its germination, hyphal elongation, and conidiation (Figures 3 and 4). Green fluorescence was detected on each developmental stage as observed in CLSM, demonstrating viabilities. The inoculated conidia on leaflets germinated at 1 dpi (Figure 3(a)). Some of the germinated conidia had ambiguous boundaries with adjacent conidia or leaf surface (Figure 4(a–c)). Conidiations were found from 2 dpi. All conidiation observed at 2 and 3 dpi were microcycle conidiation, in which conidia were produced on germ-tube tips (Figures 3(b), Figures 4(d–f)). In most cases of microcycle conidiation, a single conidium was produced on a germ-tube, and multiple conidia production was observed as infrequent (Figure 4(f)). Conidia with swollen germ-tube tips were also frequently observed, which possibly were either microcycle conidia or appressoria (Figure 4(g,h)). An enlarged structure on a germ-tube tip appeared to be an appressorium rather than microcycle conidia because the germ-tube appeared to crawl on the surface (Figure 4(h)). The proportions of germinated conidia and conidia with swollen germ-tube tips or microcycle conidia at 3 dpi were 15.47% ± 2.17% and 2.00% ± 1.31% (mean ± S.D., n = 3), respectively. The proportion of germinated conidia on a slide glass was 9.60% ± 2.01% (mean ± S.D., n = 3, supplemental Figure 1), which was lower than that on tomato leave (*p* = 0.013, n = 3, Welch t-test).At 3 dpi and later, some germinated conidia had a rough surface compared to ungerminated conidia (Figure 4(d,f)). Mucilage-like materials were also found around the germinated conidia (Figure 4(e,i)). Long hyphal developments were observed after 3 dpi around trichomes (Figure 4(j)). Multiple conidia productions from phialides that branched from elongated hypha were observed at 7 and 14 dpi (Figures 3(c,d), Figures 4(k)). Green fluorescence of newly developed hyphae and conidia, which may include ungerminated inoculated conidia, was observed in leaflets with CLSM, indicating viability until 14 dpi. Hyphae randomly elongated on the surfaces and did not enter the stomata or penetrate surfaces. Green fluorescence was not detected in the inner leaf tissues in the inspection of 30 cross-sections of inoculated leaflets by LSCM (Figure 3(e)).

## 4. Discussion

This study revealed the mode of actions of commercially important entomopathogenic fungus *A. muscarius* strain IMI 268317 using constitutive GFP-expressing strain Amgfp on tomatoes. In the stage of 0–12 hpi, the proportion of the remaining Amgfp on leaflets was increased, which suggests adhesion of conidia on tomato leaflets (Figure 1). Strengthening of adhesion of conidia on a plant surface over time has been reported with some entomopathogenic fungi, such as *B. bassiana* and *M. anisopliae* (Wagner 1997; Wang and St Leger 2007). In addition, *M. anisopliae* promotes its adhesion to plant surfaces through adhesine protein (Wang and St Leger 2007). Adherence of entomopathogenic fungi on plant surfaces seems a common phenomenon because dead insects infected by fungal pathogens are frequently observed on plants, and sometimes hyphae growing from hosts reach to the plant surfaces (e.g.*Ophiocordyceps unilateralis* in Lin et al. 2020), which may increase their fitness. However, there are only a few reports on either *Akanthomyces* spp., or their synonyms (*Lecanicillium* spp., or entomopathogenic *Verticillium* spp.). SEM observation results showed mucilage-like materials found around germinated conidia on tomato leaflets at 3 and 7 dpi (Figure 4(e,i)). Conidia with ambiguated boundaries and adjacent conidia or leaf surface found at 1 dpi may indicate mucilage secretion (Figure 4(a–c)). Rough surfaces found on the germinated conidia also suggest secretion of mucilage (Figure 4(d,f)); however, they possibly be signs of the collapse of conidia. Many entomopathogenic fungi, including *Akanthomyces*spp., produce mucilage on insect cuticles (Zacharuk 1970; Campos et al. 2005; Askary and Yarmand 2007; Güerri‐Agulló et al. 2010; Leemon and Jonsson 2012; Gao et al. 2015). However, mucilage production on plant surfaces has not been reported for entomopathogenic fungi. Mucilage produced by entomopathogenic fungi is considered to strengthen the adhesion to the host insect’s cuticle and degrade cuticles by the hydrolytic enzymes included in the mucilage (Leemon and Jonsson 2012; Butt et al. 2016). The observed mucilage-like materials in this study may also have promoted adhesion to tomato leaf surfaces.

CFUs of Amgfp on tomato leaflets consistently increased until 14 dpi for 1 × 10^5^ and 1 × 10^6^ conidia/mL inoculum (Figure 2(a,b)). Increase from 0 to 14 dpi was also confirmed for 1 × 10^7^ conidia/mL inoculum (2.4-fold increase, *p* = 0.02, n = 4, Welch t-test). Amgfp germinated at 1 dpi and started to produce conidia on germ-tube tips from 2 dpi, while general conidiation on the tip of phialides that elongated from hypha was observed at 7 dpi. Invasion into leaf tissues has never been observed. However, for *Akanthomyces* spp., long persistence on crop leaves and growth with conidiation on cucumber plants were reported (Verhaar 1997; Koike et al. 2004), while the increase of biomass on plant leaves has not been reported. The increase of the biomass of entomopathogenic fungi on tomato leaflets has also been reported for the *B. bassiana*GHA strain (Nishi et al. 2020). This multiplication implies that Amgfp can utilise nutrition on tomato leaflets. We confirmed the continuous decrease of Amgfp CFUs inoculated on a filter paper until 14 dpi despite hyphal growth (Supplemental Figure 2(a,b)), which supports the necessity of external nutrient sources to increase CFUs. The smaller increase rate of CFUs for 1 × 10^6^ conidia/mL inoculum than 1 × 10^5^ conidia/mL inoculum suggests limited nutrient sources on leaflets.

The low conidial germination at 3 dpi (15.47% ± 2.17%) suggests heterogeneous microhabitat environments on tomato leaflets, and some microenvironments on the leaflets promoted germination while the others did not. Trichomes, which are scattered on tomato plant surfaces, secrete mucous, resins, volatile oils, and other substances (Zhang et al. 2020) and can make heterogeneous microenvironments on leaflets. The conidial suspension was sprayed on leaves, which may have spread trichome secretions unevenly on the surface. Trichome secretions affect the growth of some fungi; however, their effects against entomopathogenic fungi have been unknown. For example, exudates of type IV glandular trichomes of the wild tomato *Lycopersicon pennellii*showed antifungal activity against conidia of the tomato powdery mildew pathogen *Oidium neolycopersici* (Nonomura et al. 2009). Consequently, *Periglandulaipomoeae*, which is closely related to both grass endophytes and hypocrealean entomopathogenic fungi, is a natural epiphyte specifically found on trichomes of Convolvulaceae (Steiner et al. 2011). Further understanding of responses of entomopathogenic fungi against trichome secretions and other plant surface materials will help in the better application of tomato pest control.

Amgfp produced conidia through microcycle conidiation (i.e. conidia production on germ-tube tips) from 2 dpi and general conidiation (i.e. production of conidia on tips of phialides), as observed on tomato leaflets in the later stages (Figures 3(c,d), Figures 4(d–f,k)). Although microcycle conidiation has been observed in more than 100 fungal species across various taxonomic groups, including entomopathogenic fungi (Hanlin 1994; Bosch and Yantorno 1999; Nishi et al. 2020), this is the first reported case of microcycle conidiation of *Akanthomyces* spp. and their synonyms. The entomopathogenic fungus *B. bassiana* strain GHA also produces microcycle conidia on tomato leaflets (Nishi et al. 2020). Germ-tubes with swollen tips (Figure 4(g,h)) possibly developed into microcycle conidia; however, it was difficult to distinguish them from appressoria when the tips directly contacted the leaf surface. *Beauveriabassiana*seems to have the same difficulty, as an appressorium of *B. bassiana* found on horse-chestnut leaves reported by Barta (2018) appears to be a microcycle conidium. Microcycle conidiation of Amgfp seems to have a less direct contribution to its epiphytic multiplication because the proportion of conidia with swollen germ-tube tips or microcycle conidia was only 2.00% ± 1.31%, and in most cases, only a single conidium was produced on a germ-tube tip.

Microcycle conidiation is generally induced by heat or starvation stresses and considered as a survival strategy in unfavourable environments (Hanlin 1994). *Beauveria bassiana* also induced microcycle conidiation *in vitro* by the starvation of carbon and nitrogen sources (Bosch and Yantorno 1999). Starvation or some kinds of stresses may have caused microcycle conidiation of Amgfp, considering the unfavourable conditions on tomato leaflets as inferred from its low germination rate. The best response against an unfavourable environment is not to germinate from the beginning. However, mistimed germination will occur easier in heterogeneous environments where favourable and unfavourable factors coexist. Amgfp may raise its survival rate on tomato leaflets through microcycle conidiation by avoiding useless death caused by mistimed germination.

This study has revealed that *A. muscarius* can epiphytically grow on tomato leaflets in a humid condition through conidial adhesion, germination, and general and microcycle conidiation. *Akanthomycesmuscarius* strain IMI 268317 is currently used as a mycoinsecticide in a humid greenhouse; therefore, the observed lifecycles in this study are assumed to occur in practical application. Further studies focusing on pathogenicity of newly produced propagules on leaves is necessary for developing better biocontrol strategy for tomato pests.
